# Identifying Protective Health Behaviors on Twitter: Observational Study of Travel Advisories and Zika Virus

**DOI:** 10.2196/13090

**Published:** 2019-05-13

**Authors:** Ashlynn R Daughton, Michael J Paul

**Affiliations:** 1 Analytics, Intelligence, and Technology Los Alamos National Laboratory Los Alamos, NM United States; 2 Information Science University of Colorado, Boulder Boulder, CO United States

**Keywords:** social media, travel, behavior, communicable diseases, zika virus, public health, epidemiology, information science, travel-related illness

## Abstract

**Background:**

An estimated 3.9 billion individuals live in a location endemic for common mosquito-borne diseases. The emergence of Zika virus in South America in 2015 marked the largest known Zika outbreak and caused hundreds of thousands of infections. Internet data have shown promise in identifying human behaviors relevant for tracking and understanding other diseases.

**Objective:**

Using Twitter posts regarding the 2015-16 Zika virus outbreak, we sought to identify and describe considerations and self-disclosures of a specific behavior change relevant to the spread of disease—travel cancellation. If this type of behavior is identifiable in Twitter, this approach may provide an additional source of data for disease modeling.

**Methods:**

We combined keyword filtering and machine learning classification to identify first-person reactions to Zika in 29,386 English-language tweets in the context of travel, including considerations and reports of travel cancellation. We further explored demographic, network, and linguistic characteristics of users who change their behavior compared with control groups.

**Results:**

We found differences in the demographics, social networks, and linguistic patterns of 1567 individuals identified as changing or considering changing travel behavior in response to Zika as compared with a control sample of Twitter users. We found significant differences between geographic areas in the United States, significantly more discussion by women than men, and some evidence of differences in levels of exposure to Zika-related information.

**Conclusions:**

Our findings have implications for informing the ways in which public health organizations communicate with the public on social media, and the findings contribute to our understanding of the ways in which the public perceives and acts on risks of emerging infectious diseases.

## Introduction

### Social Media for Public Health

Internet data, including data from social media platforms such as Twitter, have been used extensively in recent years to study health patterns and better understand infectious disease outbreaks [1]. Although it is known that social media usage is demographically biased [2], these data are thought to be fundamentally changing health care [3]. Social media data have been studied to provide insights into public health discourse [4,5] and concerns [6,7].

A particularly successful area of research has used internet data to improve the forecasting of disease outbreaks. Several studies have found that these data, when combined with traditional sources of epidemiological data, can improve the surveillance and forecasting of seasonal diseases such as flu [8-12] and mosquito-borne diseases such as dengue [13,14] and West Nile [15].

In this study, we have considered disease epidemics from the perspective of human *behaviors* that can affect a disease outbreak. We studied the recent outbreak of Zika virus, a mosquito-borne virus that has recently been linked to birth defects and other disorders, as a domain for studying disease-relevant behavior. We have focused on a specific behavior, decisions to change travel to avoid areas affected by Zika, because of the extensive literature that travel contributes significantly to infectious disease emergence [16,17]. We have used a combination of content analysis and supervised machine learning techniques to understand first-person accounts of travel-related decisions during the Zika outbreak. This study aimed to answer the following research questions (RQ):

1. *RQ1:* Can we identify individuals who report they changed their travel behavior in response to concerns about Zika?
2. *RQ2:* What are the characteristics of Twitter users who change or consider changing their travel behavior? In particular, we wished to know:

- *2(a):* Are there temporal, geospatial, or gender-based patterns in users who change their behavior?
- *2(b):* Are there linguistic differences in messages posted by these individuals compared with users selected at random from Twitter?
- *2(c):* Are these individuals exposed to more information about Zika on Twitter?

We have answered these questions by analyzing a collection of 29,386 English-language tweets filtered for keywords describing Zika and travel. We used a cascade of 3 machine learning classifiers to identify behavior mentions in tweets, and we have proposed a method of incorporating classifier error into our statistical analyses to test our hypotheses.

### Zika Emergence

Mosquito-borne infections have long been known to cause large outbreaks that result in substantial morbidity and mortality. An estimated 3.9 billion individuals live in a location endemic for common mosquito-borne diseases, for example, dengue, chikungunya, and now, Zika [18]. Although Zika emerged only recently in Central, South, and North America, the virus was originally discovered in 1947 in Uganda [19]. Through the 20th century, documented outbreaks were rare. The first outbreaks occurred in 2007 in Gabon and the Federated States of Micronesia [19]. Furthermore, 6 years later, French Polynesia experienced the first large outbreak, and there was a documented association between neurological symptoms and Zika [19]. The subsequent emergence of Zika in South America in 2015 marked the largest known Zika outbreak and caused hundreds of thousands of infections [19-21]. Between 2015 and 2017, the Pan American Health Organization (PAHO) reported over half a million suspected Zika cases in South and Central America [22].

For the overwhelming majority, Zika is a mild infection; the majority of cases are asymptomatic [23]. However, for some, Zika infection can lead to more serious complications, including the neurological syndrome, Guillain-Barré [24], and birth defects in fetuses infected in-utero [25].

Importantly, these causal relationships have only been recently established. In October 2015, Brazil reported an association between Zika cases and microcephaly, a condition where an infant’s head circumference is extremely small and is accompanied by severe developmental and health complications [26], and others noted a possible association with Guillain-Barré syndrome in adults [24]. As evidence mounted that there was a causal relationship, the World Health Organization (WHO) and PAHO issued alerts in December 2015 about the association between Zika, neurological syndromes, and birth defects. The United States responded to these alerts in mid-January 2016 by issuing a travel advisory for pregnant women, which cautioned against traveling to locations with local Zika transmission [21]. Zika was then declared a public health emergency by the WHO in February 2016 [21].

### Travel and Infectious Disease

Travel advisories are an important public health intervention because of the documented impact of travel on the emergence of infectious diseases [16,17]. Historical case studies describe imported cases of diseases that led to large outbreaks as early as the 1500s [16]. In the present day, there are many outbreaks that have been attributed to travel from other regions. For example, genetic data from the 2009 H1N1 outbreak show that the movement of swine around Mexico was responsible for outbreaks in various provinces [27]. Genetic evidence further indicates that H1N1 was probably introduced to the United States from both Mexico and Asia [27].

Simulations find that the impact of travel on disease spread varies based on a number of factors. For example, Bajardi et al found that travel restrictions could reduce cases but probably only minimally [28]. However, research done by Huizer et al finds that air travel could have dramatically changed the 1968 pandemic influenza in Hong Kong [29]. In general, travel is thought to play an important but variable role in disease transmission. Current recommendations are to implement travel-related control measures as necessary [30,31].

### Social Media and Zika

Internet data have been used to better understand individual health behaviors and health discourse on the Web. Studies have found evidence that users publicly discuss a variety of ailments [4], as well as particular behaviors used to prevent ailments. For example, Signorini et al observed discussions of behaviors such as hand washing and wearing masks to prevent the flu [10]. Paul and Dredze similarly note that individuals often report medications used for symptom relief [4].

As the largest known Zika outbreak occurred recently, researchers are only now beginning to investigate the use of internet data to understand this particular disease. McGough et al used an autoregressive modeling approach to combine epidemiological data from PAHO, Twitter, Google search queries, and reports from HealthMap to build short-term forecasts for several Central and South American countries. They found that the lowest error models were produced when using Google search query volumes [32].

Others have found important information in Twitter data. Stefanidis et al used tweets from the first 3 months of the outbreak to characterize discourse around Zika [33]. These data were used to look at the emergence of spatial clusters in online discussions of Zika on Twitter and to identify distinct geospatial communities that participated in the conversation early in the outbreak. In particular, they found that Twitter users tended to use public health organizations to find information and did not generally use Twitter as a way to interact with organizations directly [33]. Using data encompassing more of the outbreak, Miller et al used Twitter to identify tweets about treatment, transmission, and prevention of Zika and noted the use of Twitter as a way to monitor concerns in the general population [34].

Sharma et al investigated information dispersion on Facebook and specifically noted that inaccurate or misleading posts were more popular than those with scientifically sound information [35]. This observation is consistent with previous work which identified rumors and health misinformation on Twitter [36]. Similarly, Gui et al noted that even official sources of information were unreliable during the outbreak because of incomplete information and observed that the internet provided spaces that allowed individuals to frame risk and decisions [37].

Seltzer et al used Instagram to look at image-sharing practices around Zika [38]. They found that health-related images related to Zika were predominately about transmission and prevention and suggested that Instagram could be used to track sentiment with regard to Zika [38].

### Motivation and Contributions

Zika is likely to continue to be an emerging illness of concern with considerable impacts in South, Central, and North America [39]. In contrast to previous work that has focused on the discourse on different platforms or the possible utility of various internet data sources for modeling forecasting, this study focused on identifying a particular behavior of impact on the spread of the disease—travel change. As a first step, this study aimed to identify individuals on Twitter who chose to change their behaviors (RQ1), understand the characteristics of those individuals (RQ2a), and test explanations for any patterns observed (RQ2b and 2c).

Human behaviors directly impact disease transmission [40,41]. Previous work has found that travel patterns are important for transmission but difficult to quantify because of a general lack of available data [41]. A long-term goal of this study is to incorporate behavior change data into disease-forecasting models. This initial study focused on the important first step of identifying travel behaviors and characterizing the factors that affect them.

## Methods

**Figure 1:**
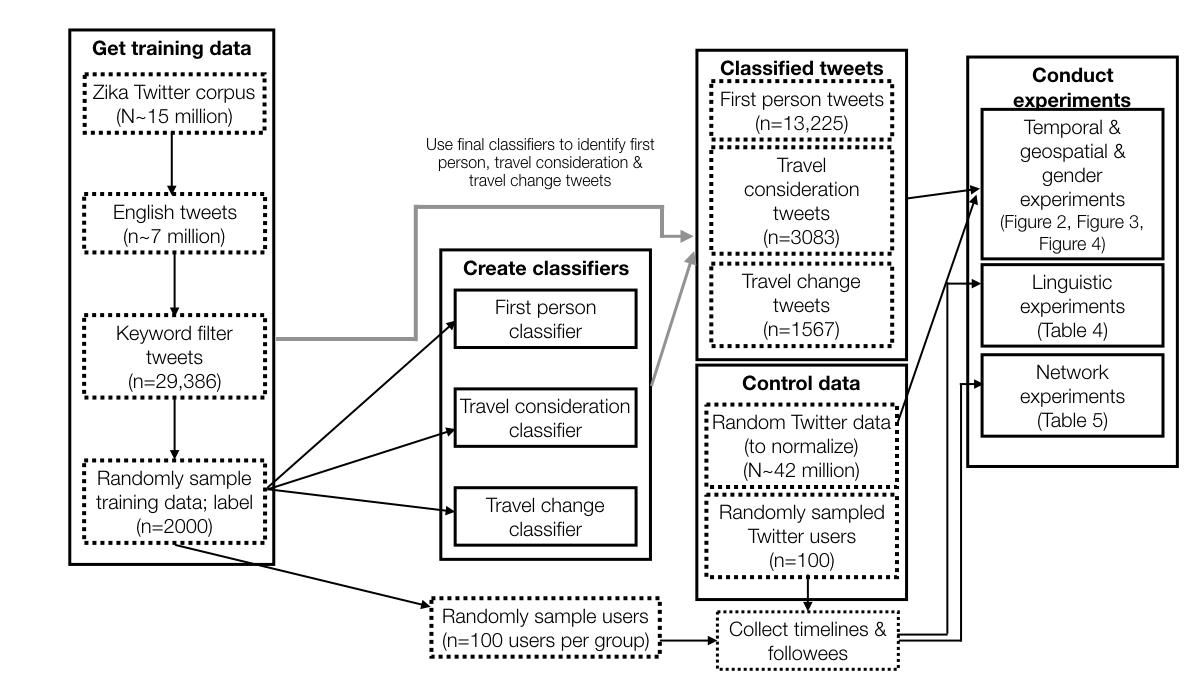
Data processing and experimental overview. Dotted boxes show datasets and corresponding sizes where applicable. Solid boxes show methods used and reference relevant text figures or tables. Black arrows show the flow of data through the pipeline. The gray arrows denote that the final classifiers were used to identify first person, travel consideration, and travel change tweets from the keyword filtered tweets.

This section describes the process used to identify relevant tweets and the techniques used to train and tune the classifiers. We then provide details on the collection of the Twitter timeline and followee data used in later analyses. Figure 1 summarizes the various datasets and methods.

### Identifying Relevant Tweets

Our data come from a set of 15 million Zika-related tweets from March 1, 2015, to October 31, 2016, with about 7 million in English, described in Daughton et al [42]. This collection includes all tweets mentioning *Zika* and related terms during this time period.

Qualitatively, we observed that the bulk of these Zika tweets were sharing news or other information, usually with links to external articles. However, we also observed a number of English-language tweets describing personal or shared experiences with Zika, including behavior changes in response to concerns about Zika (eg, changing travel plans or buying a mosquito repellent). This section describes our approach to identifying such personal mentions of travel-related behavior through a pipeline of keyword filtering and supervised machine learning.

### Keyword Filtering

As personal mentions of travel behavior are a very small proportion of the dataset, we first filtered the dataset to provide a subset with a higher fraction of relevant tweets. This is a standard approach in many social media applications to obtain a large enough fraction of relevant instances to build a reasonably balanced training set [43-47]. In our study, tweets were filtered for those that contained (1) at least one of a set of first-person pronouns to target personal behaviors and (2) at least one of a large set of travel-related terms (see Multimedia Appendix 1).

To be as comprehensive as possible when constructing the list of travel-related terms, we included all major airlines in the United States and all airlines with flights to South America [48,49]. Twitter handles of the airline, cruise, and travel agency companies, including official Twitter handles as well as handles used for negative feedback, were included. These were manually curated by searching for the company on Twitter and identifying associated handles.

After filtering and excluding retweets, 29,386 English-language tweets matched these criteria.

### Classification

After keyword filtering, we still observed a variety of tweet topics in the data. This included mentions of changes in travel, opinions about the Olympics (which were hosted in Brazil during the outbreak), opinions about quarantining travelers, and general worry about Zika. The filters also captured tweets that were neither first person nor about travel, such as the headline, *Spraying Mosquitoes by Plane Ain’t Perfect, But It’s the Best We’ve Got for Zika - WIRED*.

To further filter the dataset to tweets of relevance to this study—tweets in which people express that they are personally changing or thinking about changing their travel behavior—we constructed 3 binary classifiers:

1. *First person:* Tweets where someone makes their own comment related to Zika in contrast to sharing external content. This can include jokes, opinions, observations, and questions. This category does not include headlines, promotion or solicitation for articles or events, or generic requests for congresspersons to fund Zika.
2. *Travel consideration:* First-person tweets that are about the tweeter’s travel plans. This can include tweets that explicitly express the desire to change or not change travel, as well as tweets that are concerned but undecided about travel change.
3. *Travel change:* Travel consideration tweets that explicitly indicate that the tweeter has changed travel plans or is actively trying to change their travel plans. We also attempted to categorize tweets that explicitly said the user would not change travel, but we were unable to build a reliable classifier (F1=.35) and, therefore, excluded it from this study. Messages such as *I want a refund for my trip* would be labeled as travel change whereas messages such as *I’m interested in your refund policy* would not.

Each category only applies to tweets positively labeled with the previous category—travel consideration tweets must also be first-person tweets, and travel change tweets must also be travel consideration tweets.

### Annotation

To create a training set for learning supervised classifiers, we randomly sampled 2000 English-language tweets from the keyword-filtered dataset and annotated them with the 3 categories above. Furthermore, 2 researchers independently annotated all tweets to measure agreement. As tweets were only labeled for travel consideration and travel change when they were labeled with the previous category, we only calculated agreement for these categories when annotators also agreed on the previous category. This can be interpreted as measuring: in the cases where annotators agreed on first person, what was their agreement on travel consideration?

Examples of each category, frequency, and agreement are shown in Table 1. To create the final set of labeled data, the 2 annotators discussed the disagreements and updated category criteria to resolve disagreements. For example, annotators disagreed on whether promotion or solicitation of articles or events, as well as requests for congresspersons to fund Zika should be in the first-person category. After discussion, we clarified the criteria to exclude those types of tweets. Using these updated criteria, disagreements were resolved, and the final labels were selected.

**Table 1:** Label frequency (%), annotator agreement (Cohen’s κ), and example tweets for each classification category.

Category	Example (paraphrased)	% (n/N)	κ
First person	When Zika explodes after the Olympics, I’m going to say I told you so!	41.15% (823/2000)	.52
Travel consideration	Thinking about going to Rio for honeymoon. Will I be safe with Zika?	17.5% (350/2000)	.76
Travel change	So mad I had to cancel my island babymoon because of Zika	10.8% (216/2000)	.66

### Training and Evaluation

All classifiers were binary logistic regression classifiers built using the Python package scikit-learn (version 0.19.1) [50], where the 3 classifiers were used in a pipeline. Binary logistic regression is an attractive method because outputs are easily interpretable and can be easily tuned for optimal precision and recall. Furthermore, this is a common method used in other health surveillance work [9,51]. Twenty percent (400/2000) of the initial dataset was reserved for testing. This is a standard method used in machine learning to avoid overfitting models [52]. On the training data, we used a grid search to learn the best regularization parameter and feature set, using 5-fold cross-validation to measure the validation performance. For all classifiers, we tested features that included 1-, 2-, and 3-grams. Unigrams (1-grams) consistently outperformed longer n-grams or combinations of n-grams. We also experimented with feature selection using a chi-square test in an attempt to improve classifier metrics [53]. The best results were obtained when all features were used (first person and travel consideration) and when the top 70% of features were used (travel change; see Multimedia Appendix 2). Tweets were preprocessed to remove emojis, punctuation, and consecutive identical characters (eg, vowel elongation) and to replace URLs and usernames with generic tokens.

Performance results on the held-out test data are shown in Table 2. Note that the F1 values shown here differ from those shown in Multimedia Appendix 2 because Multimedia Appendix 2 was generated using cross-validation on the training data, whereas the final metrics were generated using the testing data. We observed that the travel consideration classifier performs the weakest. We also compared the pipeline approach with stand-alone travel consideration and travel change classifiers. However, this method resulted in significantly worse F1 scores (.63 and .65, respectively), and thus, we proceeded with a pipelined approach. The next subsection describes how we account for the cascade of classifier errors in our statistical analyses.

Precision is a measurement of type I error and describes the number of selected items that are actually relevant (percent of those classified positive that are actually positive). Recall, related to type II error, instead describes how many relevant items are selected (percent of positive instances in the full dataset that are classified positive). F1 then combines these 2 metrics, using a harmonic mean, to describe the system overall. We show both F1 using the pipelined approach (the final classifier) as well as the F1 score if each classifier is built independently (see Table 2).

**Table 2:** Final precision, recall, and F1 of the 3 classifiers.

Classifier	Precision	Recall	F1	F1 (no pipeline)
First person	0.89	0.94	0.92	0.92
Travel consideration	0.61	0.74	0.67	0.63
Travel change	0.66	0.81	0.73	0.65

### Statistical Analysis

Our analyses involve measuring the proportion of tweets classified as the various categories along different dimensions. When appropriate, we have provided CIs of these estimates. Our CIs are based on *bootstrap resampling* [54], a nonparametric technique that works as follows. A single bootstrapped estimate of the desired statistic (eg, proportion of tweets) is estimated by resampling the dataset with replacement (bootstrapping) and calculating the statistic from the randomly sampled version of the dataset. This is repeated many times (1000 times in our experiments) to construct a distribution of bootstrapped estimates, and the middle 95% of the estimates are taken as a 95% CI for that statistic [55].

We further modify this approach to account for the uncertainty present in the classifier, using the negative predictive value (NPV) and the positive predictive value (PPV). The NPV is the ratio of true negatives to the sum of true negatives and false negatives whereas the PPV (equivalent to precision in classification) is the ratio of true positives to the sum of true positives and false positives (see [56] for an extensive description of the method). By using this method, we are able to account for the inaccuracies of the individual classifiers and avoid propagating error through the pipeline. We refer to this method as a *weighted bootstrapped CI* in all relevant figures.

### Timeline and Followee Collection

Owing to the widespread attention the Zika outbreak received in the media, we wanted to identify if there are other characteristics that differentiate users who changed or considered changing travel as compared with users who tweeted about Zika but did not discuss travel plans.

Using our labeled training data, we collected a set of 100 users sampled at random for each of the 3 classification categories. To construct comparison groups, we also sampled 100 users from the entire set of English-language Zika tweets, as well as 100 English-language users selected at random from all of Twitter. When sampling, we excluded verified users, as the inclusion of celebrities and other prolific accounts could bias the results. We then identified 3 sets of 100 users at random for each classifier. For each group, we collected the Twitter timelines of the users and the list of individuals they follow (their *followees*) using Tweepy [57]. These data were downloaded in January 2018.

Owing to Twitter’s application programming interface (API) restrictions on user timelines, we were only able to collect the most recent 3200 tweets for each user. This means that we were not able to collect tweets during the time period of the Zika outbreak especially frequently. This could affect the analyses but will be a close approximation as long as these users have not substantially changed their tweeting behavior since 2016. Tweets were preprocessed in the same manner as described in the Classification section.

## Results

Applying the classifiers to the keyword-filtered tweets resulted in a final dataset of 13,225 first-person tweets, 3083 travel consideration tweets, and 1567 travel change tweets. This section describes the results of our analyses of these tweets and the users who posted these tweets.

### Temporal Patterns

**Figure 2:**
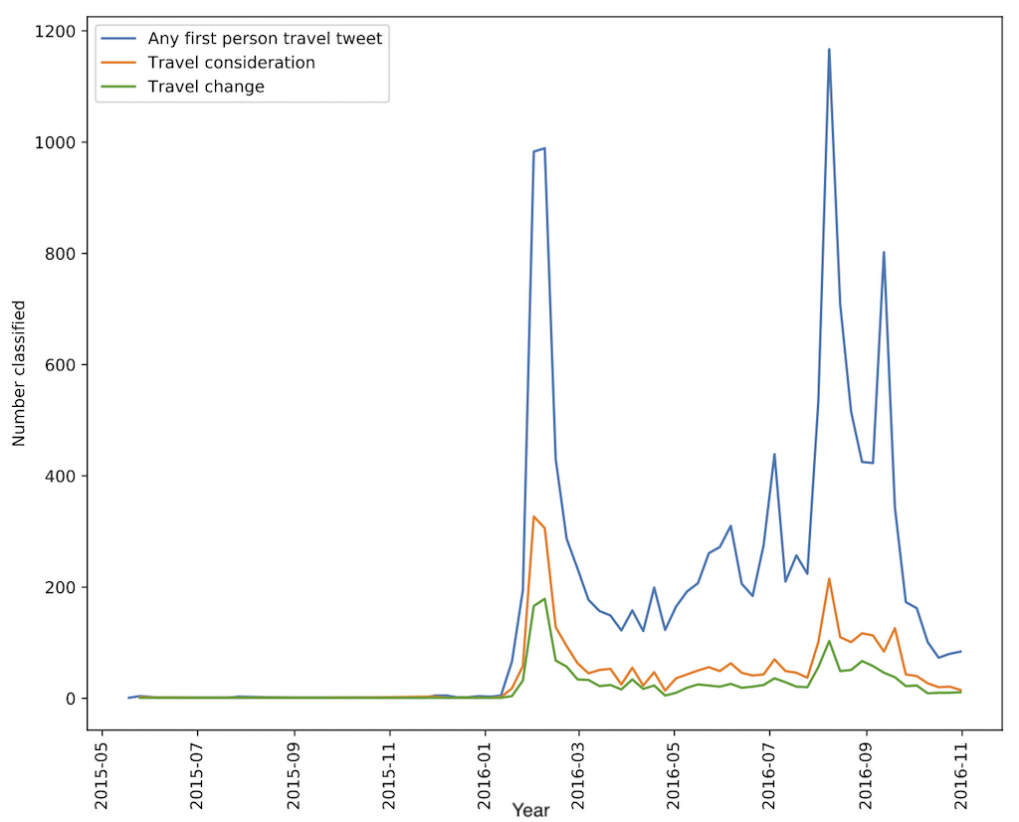
Temporal trends in classifications by week.

Temporal trends in the 3 datasets are shown in Figure 2. Two major spikes corresponding to important events during the outbreak are evident. The first occurred in February 2016 during the time of initial travel advisories by the WHO and the Centers for Disease Control and Prevention [21]. The second, more gradual peak occurs in the summer of 2016 and appears to correspond to the summer Olympics in Rio de Janeiro. We noticed an increase in travel change tweets primarily during the initial set of travel advisories, rather than sustained interest in travel throughout the course of the outbreak.

**Figure 3:**
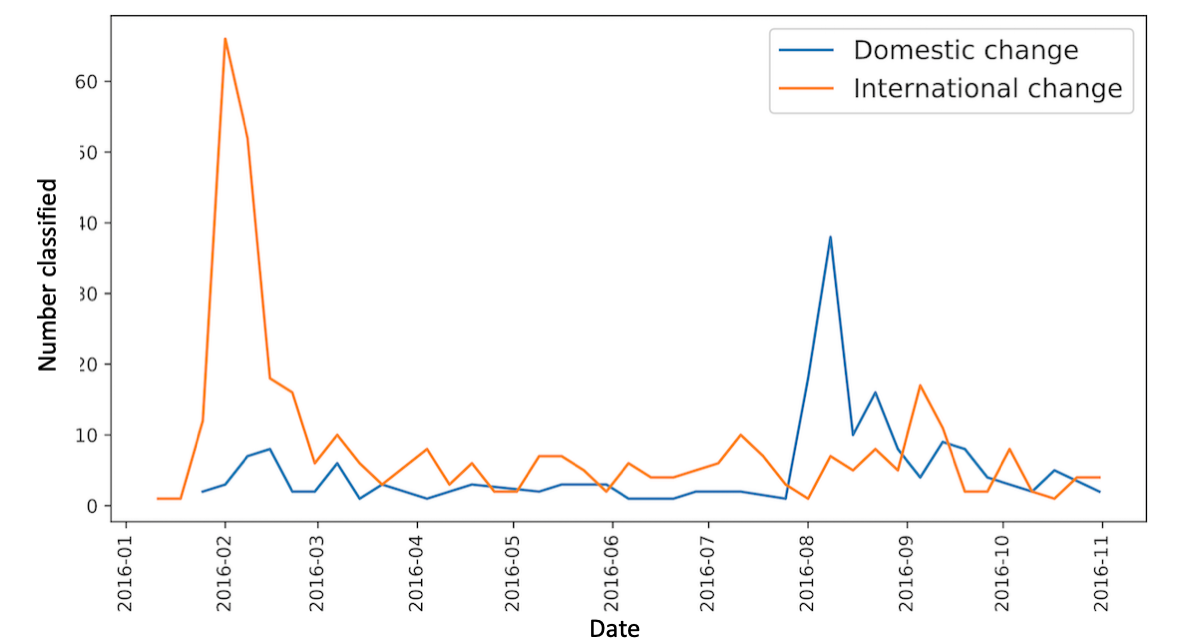
Temporal trends in decisions to change international (outside of the United States) and domestic (within the United States) travel.

We also explored temporal differences in the destinations of the users’ cancelled travel. To do this, we manually labeled the destinations in all 1567 tweets that were classified in the *travel change* category as international or domestic with regard to the United States. Many tweets were not specific about the location of travel plans; we were able to identify 34% of *travel change* destinations. We found 2 distinct peaks in decisions to change travel (Figure 3). International change spikes sharply in conjunction with the initial travel advisories of February 2016, whereas domestic change spikes sharply in August 2016. The latter spike aligns in time with evidence of local Zika transmission in Florida that was first identified in July 2016 [58] and may also correspond to the increase in cases in US territories such as Puerto Rico [59]. There is an additional peak in the international change tweets in September 2016. These tweets primarily discuss canceling travel to Singapore, which had started to identify local cases in late August 2016 [60]. Although the volume of tweets is small, they show a timely response to the news that Zika had emerged and was circulating locally, within a week of the initial official Ministry of Health report [60].

### Geospatial and Gender Patterns

#### Geospatial Variability

To evaluate spatial trends, we geolocated tweets using Carmen [61], which resolves tweets to structured locations using geographic coordinates when available and user profile information if not.

We grouped tweets into geographic regions defined by the US Department of Health and Human Services (HHS). HHS Regions are regional groupings of states in the United States that are commonly used to aggregate states for health studies. As the traditional HHS Regions group geographically disparate states together (eg, Hawaii and island territories are grouped with mainland regions), we modified the HHS Regions as follows:

1. R1: Connecticut, Maine, Massachusetts, New Hampshire, Rhode Island, Vermont.
2. R2: New Jersey, New York.
3. R3: Delaware, District of Columbia, Maryland, Pennsylvania, Virginia, West Virginia.
4. R4: Alabama, Florida, Georgia, Kentucky, Mississippi, North Carolina, South Carolina, Tennessee.
5. R5: Illinois, Indiana, Michigan, Minnesota, Ohio, Wisconsin.
6. R6: Arkansas, Louisiana, New Mexico, Oklahoma, Texas.
7. R7: Iowa, Kansas, Missouri, Nebraska.
8. R8: Colorado, Montana, North Dakota, South Dakota, Utah, Wyoming.
9. R9: Arizona, California, Nevada.
10. R10: Alaska, Idaho, Oregon, Washington.
11. Caribbean Islands: Puerto Rico, US Virgin Islands.
12. Pacific Islands: Hawaii, American Samoa, Northern Mariana Islands, Federated States of Micronesia, Guam, Marshall Islands, Republic of Palau.

We ultimately excluded both the Pacific Islands and Caribbean Islands from this analysis because there were not enough tweets classified in these regions (fewer than 50 tweets each).

As tweet volume varies by location, we created a type of per-capita estimate to adjust for the overall popularity of Twitter in each region. We collected a 1% sample of tweets from the Twitter streaming API over approximately 10 nonconsecutive days throughout December 2017 and January 2018 to normalize the estimates (42.1 million tweets). The number of tweets classified from each region was then divided by the total number of tweets from that region in the random sample.

**Figure 4:**
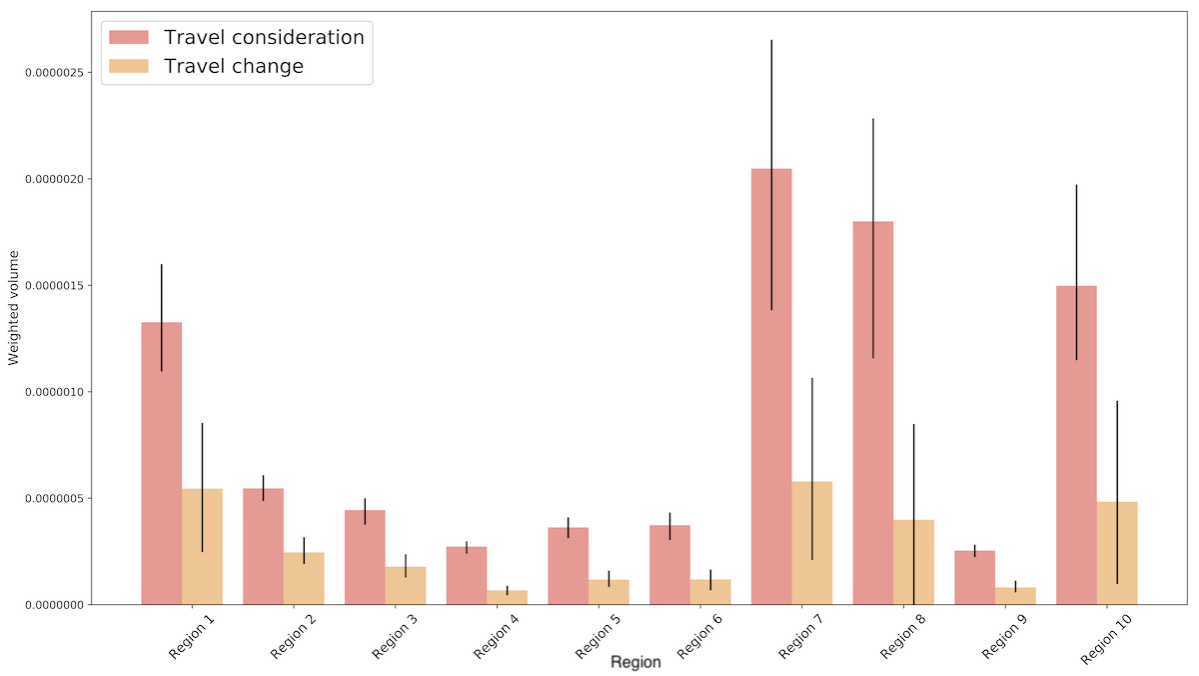
Weighted volume of classified tweets by modified US Department of Health and Human Services Region. Bars show median weighted volume. Error bars represent 95% confidence intervals obtained using weighted bootstrapped sampling.

Figure 4 shows a wide variation in the weighted volume of tweets across different spatial regions of the United States. Regions 1, 7, 8, and 10 have the highest relative volume of tweets considering and changing travel plans. These regions predominantly consist of landlocked states in the center of the country and include individuals who would have only been at risk of Zika infection if they traveled to an area with local transmission. Interestingly, regions that included states where Zika transmission occurred (Florida—Region 4 and Texas—Region 6) were among the lowest in weighted volume of tweets. It could be that individuals in these locations were not tweeting about travel change because they were at a more acute risk of infection. It is also possible that more granular (eg, state-level) observations are obscured by aggregation to the HHS level.

#### Gender Variability

**Figure 5:**
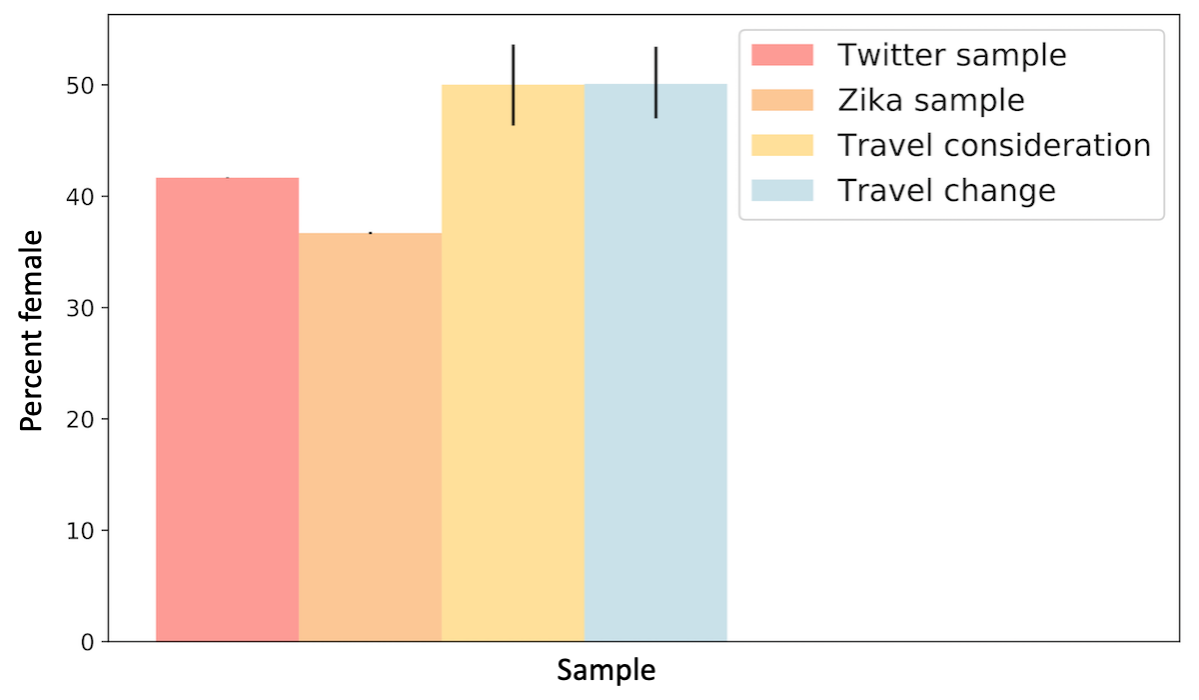
Relative percent of women in a sample of Twitter (red), English Zika dataset (orange), travel consideration dataset (yellow), and the travel change dataset (blue). Bars show 95% weighted bootstrapped confidence intervals.

As Zika is primarily a concern for women who are pregnant or trying to become pregnant, we investigated the relative percentage of women tweeting versus men (Figure 5). Gender was inferred using the *Demographer* tool [62], which infers gender of Twitter users with an estimated 94% accuracy based on character n-grams of the persons’ names.

### Linguistic Comparison

To better understand the factors that contribute to a decision to change travel, we compared the style and content of messages between users in the travel consideration and travel change groups with the random sample of Twitter users. We hypothesized that those who discuss Zika travel are more likely to talk about health in general than typical Twitter users and that those who consider changing travel may have higher levels of fear or anxiety.

We used Linguistic Inquiry Word Count (LIWC) [63], which maps various English-language terms to linguistic and psychological constructs. We selected a subset of LIWC categories related to our hypotheses (health and anxiety), as well as categories related to various personal concerns as a way of categorizing other general content of discussion. We also created a category specifically for pregnancy- related terms, using the regular expression pregnan*, because of the relevance of Zika to a developing pregnancy. Although pregnancy is included in the LIWC biological processes category, that category is much broader than pregnancy specifically.

For each user timeline and each LIWC category, we calculated the percentage of tweets that contain a term from the category. In this calculation, we excluded the tweets mentioning Zika so that the analysis does not reflect the same data used to select users. In addition, we restricted the analysis to timelines with a minimum of 10 tweets across the timeline. Finally, for each category, we calculated the average percentage across all timelines in each user group. The results are shown in Table 3.

Compared with a random sample of Twitter users, users who tweeted about changing or considering changing travel in reaction to Zika are significantly more likely to use past and present tense, as well as terms indicating social processes, perhaps indicating increased planning. Travel consideration users are significantly more likely to use personal pronouns and singular first-person pronouns and were significantly higher in the anxiety category. Travel change users were significantly more likely to use plural first-person pronouns, had higher inhibition, and tweeted more about pregnancy. There are no significant differences between the travel consideration and travel change groups.

Contrary to our expectations, the travel groups do not tweet significantly differently from the overall Twitter population about health or bodily functions. This indicates that the users we identified as part of this behavior change pipeline were uniquely concerned about Zika and did not appear to be generally more aware or interested in discussing health-related topics on social media (with the important exception of pregnancy). It would be useful to explore more on this line of inquiry in future work, as understanding who talks about infectious diseases (and how) is of immediate interest to the disease surveillance community [64].

**Table 3:** Average percent of Linguistic Inquiry Word Count category prevalence per group.

Type	Category	All Twitter	Consideration	Change
Linguistic processes	Personal pronouns	0.6080	*0.7495* ^a^	0.7501
Linguistic processes	1st singular	0.2788	*0.3673* ^a^	0.3214
Linguistic processes	1st plural	0.0458	0.0699	*0.0895* ^a^
Linguistic processes	3rd singular	0.0692	0.0699	*0.0895* ^a^
Linguistic processes	3rd plural	0.0474	0.0561	0.0571
Linguistic processes	Past tense	0.1794	*0.2538* ^a^	*0.2665* ^a^
Linguistic processes	Future tense	0.0648	0.0842	0.0871
Linguistic processes	Present tense	0.6053	*0.7711* ^a^	*0.7947* ^a^
Psychological processes	Social processes	0.7181	*0.8867*	*0.9760*
Psychological processes	Affective processes	0.6648	0.7362	0.7587
Psychological processes	Positive emotion	0.4323	0.5106	0.5105
Psychological processes	Negative emotion	0.2290	0.2225	0.2440
Psychological processes	Anxiety	0.0246	*0.0364* ^a^	0.0331
Psychological processes	Tentativeness	0.1556	0.2019	0.2075
Psychological processes	Certainty	0.1203	0.1437	0.1375
Psychological processes	Inhibition	0.0470	0.0633	*0.0680* ^a^
Psychological processes	Biological processes	0.2230	0.2712	0.2401
Psychological processes	Body	0.0705	0.0787	0.0674
Psychological processes	Health	0.0495	0.0744	0.0734
Psychological processes	Sexual	0.0857	0.0648	0.0526
Other (non- Linguistic Inquiry Word Count)	Pregnancy	0.0004	0.0106	*0.0016* ^a^

^a^Instances where there are significant differences from the random sample. Significance is estimated using an unpaired 2-sided *t* test with a significance level of *P*<.05 after Bonferroni correction.

### Network Comparison

As a final experiment, we look at the number of followees each of the randomly selected users had that were also present elsewhere in the Zika dataset—that is, the accounts a user follows that had at least one Zika-related tweet. Table 4 shows the number of Zika followees in each group as well as the number of tweets those followees tweeted that were about Zika. We calculate both the raw counts as well as normalized counts in which we divide the number of Zika followees and number of Zika tweets by each user’s total number of followees. This allows us to measure both the raw number of Zika tweets an individual could have been exposed to and the relative likelihood of exposure based on the proportion of their feeds that contained Zika content.

Although it is impossible to replicate Twitter’s algorithm for showing information on the timeline, we have the unique capability to look at network effects because we have 100% of the tweets during the time period that explicitly mentions either *zika* or *zikv*. We reasoned that individuals who have many followees who appear in the Zika corpus (ie, they follow accounts that are also tweeting about Zika) were more likely to have tweets about Zika appear in their feed. If we were to find that individuals who follow many accounts that appear in the dataset are more likely to appear in the travel change group, we would then further question the role that Twitter plays in catalyzing and informing decisions about behavior change.

Indeed, we did find that those individuals who considered or changed their travel plans had a higher number of followees and tweets that they could have been exposed to in the sample. Although the travel groups had higher counts under every metric when compared with the control group, the difference is only significant under the normalized metrics.

**Table 4:** The number of followees an individual user has who are also in the dataset, and the number of tweets that followees tweeted that are also in the dataset. We normalized to the number of total followees for each individual. Values in italics are significant (*P*≤.05).

Metric	All Twitter, median (95% CI)	Consideration, median (95% CI)	Change, median (95% CI)
Number of followees (raw)	92.8 (58.3-135.4)	111.6 (71.1-170.9)	122.2 (82.3-177.4)
Number of followees (normalized)	0.08 (0.06-0.11)	*0.15 (0.12-0.17)*	*0.17 (0.14-0.20)*
Number of tweets (raw)	93.6 (56.2-141.2)	111.3 (67.7-169.8)	122.7 (79.6-179.0)
Number of tweets (normalized)	1.71 (1.02-2.62)	*5.7 (3.41-8.74)*	*7.99 (3.47-14.98)*

## Discussion

### Principal Findings

In an age where infectious diseases are emerging and re-emerging rapidly [65], the ability to identify groups of individuals who might be at increased risk of contracting or contributing to the spread of infection can inform methods of risk communication, infectious disease interventions, and policies at a broader level.

We present supervised classifiers that identify evidence of behavior changes with regard to concerns and changes in travel plans owing to Zika on Twitter. Although previous work has observed that individuals mention protective health behaviors on social media [10], to the best of our knowledge, this is the first work to study a specific behavior change in depth. We examined temporal and demographic patterns in travel behaviors, as well as psycholinguistic markers and information exposure (as approximated through lexical and network analyses, respectively) of individuals changing behavior compared with a randomly sampled control group. More concretely, we considered 4 research questions. Their respective conclusions are discussed below.

*RQ1: Can we identify individuals who report they changed travel behavior in response to Zika?* We conclude that tweets about changing travel and considering changing travel can be identified with high recall. Furthermore, we are able to account for the comparatively lower precision achieved here using our weighted bootstrap resampling method.

*RQ2(a): Are there temporal, geospatial, or gender-based patterns in users who change their behavior?* We observed temporal patterns in travel consideration and travel change tweets, including the destination of travel, which correspond to important events in the Zika outbreak. We are encouraged that temporal trends correspond with events that we would expect to be reflected in this data stream.

We additionally find significant differences in the gender distribution of users tweeting about travel consideration and change compared with the general population of Twitter. In particular, we find that the relative proportion of women engaging in conversation indicating travel change behaviors on Twitter is higher than men. This, in combination with the results of RQ2(b) discussed below, is evidence that pregnancy was playing a role in these considerations.

For comparison with existing knowledge on this subject, we discuss 2 small surveys (85 and 121 participants) conducted in New York (NY) [66] and Miami [67] about the knowledge around Zika and travel and included related questions about behaviors. In NY, researchers found that roughly a third of women surveyed were not aware of the travel advisories in place during their travel, almost half were not aware that Zika was being transmitted in the location that they traveled to, and a relatively large number (about one-third) did not know they were pregnant at the time of travel [66]. In Miami, the vast majority of respondents were aware of Zika and reported that they changed their behaviors to avoid the disease; however, only 27% were aware that they were at risk of infection where they lived [67]. Although these survey data exclude men, they do find evidence that women were aware of the disease and that some women (though not all) took measures like changing travel plans to avoid exposure to the disease. Indeed, these surveys highlight the importance of more work in this area to further understand behavior changes over larger spatiotemporal regions.

*RQ2(b): Are there linguistic differences in messages posted by these individuals compared with users selected at random from Twitter?* We found that users in the travel categories do not appear to tweet more often about health than Twitter users in general. However, travel change users do tweet more often about pregnancy, which suggests this may be a factor in considering travel changes. In addition, travel consideration users tweet words associated with anxiety more than the general population.

*RQ2(c): Are individuals who change their behavior exposed to more information about Zika?* Travel consideration and change users have a statistically higher fraction of Zika-related followees and tweets in the sample, indicating that these users had greater exposure to Zika-related information. This is evidence that exposure to information about Zika may play a role in this decision-making process.

### Limitations

There are several limitations of the data and our methodology that must also be considered. First, it is known that Twitter is a demographically biased data source [68] and as such may not be representative of the broader population. However, this research contributes to the vast literature that uses the Twitter platform to understand health behaviors [4,10,69]. The data are additionally biased in that data only includes tweets in English, which are predominately from the United States. However, we note that data from the United States are appropriate for studying travel behaviors because there was minimal Zika transmission in the United States, as the mosquito vector is absent in the majority of the country. As such, the main method of exposure was through travel to locations with local transmission. We believe our framework could be applied to other behaviors that are only applicable in places with local transmission, such as the use of mosquito repellent, but the classifiers would need to be trained in other languages such as Latin American Spanish.

Second, we recognize the lack of external validity owing to the absence of comparable ground truth data. We view this as a motivation for this research, where findings from this study can be viewed as hypotheses to test with future experiments. It is well known that human behaviors directly impact disease transmission [40,41,70]. For example, Lau et al find that the Severe Acute Respiratory Syndrome epidemic changed individuals' travel patterns [71], and substantial research has shown that beliefs and behaviors about vaccinations dramatically impact disease occurrence [72]. However, travel-related research data are currently sparse [41]. Although we cannot say that the findings from this research are generalizable, the fact that they exist on Twitter is evidence they do exist. As such, these data can be viewed as motivations for larger survey experiments to confirm the findings and to evaluate if Twitter is a viable alternative data stream. Future work could also aim to validate behavior estimates indirectly by verifying their utility in an external task such as disease forecasting.

Third, machine learning classifiers introduce error [73], which could be further amplified by using a pipeline approach. However, we use weighted bootstrap sampling to appropriately account for these errors in downstream analyses. As our results showed significant differences even after accounting for errors, we did not make it a priority to build the best possible classifier in this work, but instead relied on standard tools.

Finally, there are some limitations of our labeled dataset. It is relatively small compared with some previous work. We specifically chose not to scale up the annotation process with crowdsourcing [74] so that the annotations were done by researchers with domain expertise. However, it is possible that a larger training set could lead to better classifier performance. Similarly, our ability to identify statistically significant differences between user groups is limited by having only 100 timelines per group. However, the rate limits of the Twitter API make it difficult to collect large numbers of user timelines. Furthermore, although small sample sizes may affect the power of the analyses, this does not affect the correctness of the approach, which correctly constructs CIs.

In addition, the labeling criteria we used could introduce bias. In particular, we can only capture people who explicitly state that they are canceling travel and that they are doing so because of Zika. Research in this field is limited, but initial work on self-reports of cold and flu illness indicates that it is rare for individuals to tweet about their health concerns [77], and it is currently unknown how this could bias the distribution of labels. However, the experiments presented here do not try to measure overall levels of travel cancellation because of these issues. Instead, we focus on comparisons across groups, which are valid if these data biases are consistent across groups (eg, gender and geography).

### Implications

The results of this study show that people do describe first-person behavior changes on Twitter and that such tweets can be classified and analyzed at scale. In particular, we find that our behavior change classifier produces a dataset that corresponds to events during the outbreak and shows evidence of geographic and gender-based differences in the behavior change.

These data support hypotheses that social media can play a role in an individual’s health choices. Other research has shown that an important predictor of population health is knowledge and that this knowledge can be disproportionate across different geographical areas based on access to health care expertise [75]. Research on ways in which social media can facilitate promotion of accurate and important health messages, thus, has clear applications.

Eventually, we envision these types of algorithms being used within the disease surveillance community. There is substantial previous work using internet data to gather traces of information about individuals’ health to monitor and forecast infectious disease outbreaks (eg, search query volumes used for Google Flu Trends). In principle, social media–derived data about behaviors that affect the spread of disease could be incorporated into forecasting models to better describe disease transmission dynamics. As part of this study, we plan to eventually incorporate this type of data into such models.

In addition to monitoring and forecasting, data and conclusions from studies such as this work can inform preventative health messaging. Previous research has found that the ways infectious diseases are framed contribute in important ways to the public perception of the event’s severity [76]. Gui et al describe the way in which individuals frame their personal risk from Zika amid uncertain or unclear public health recommendations [37]. They noted that even official sources of information were unreliable during the outbreak because of incomplete information and observed that the internet provided spaces that allowed individuals to frame risk and decisions [37]. We qualitatively observed in our data that there were many instances of individuals who were at low risk of complications resulting from Zika but were highly concerned about their personal risk from Zika. Future work in understanding how individuals frame personal risk from infectious diseases could contribute to our understanding of ways to improve public health risk communication.

## Appendix

Multimedia Appendix 1 Travel-related keywords used to filter tweets.

Multimedia Appendix 2 Cross-validated F1 scores for classifiers stratified by n-gram range and percentage of features used (based on chi-square).

## Abbreviations

API: application programming interface
HHS: US Department of Health and Human Services
LIWC: Linguistic Inquiry Word Count
NPV: negative predictive value
NY: New York
PAHO: Pan American Health Organization
PPV: positive predictive value
RQ: research question
WHO: World Health Organization
