# Supplementary material for: Identifying Protective Health Behaviors on Twitter: Observational Study of Travel Advisories and Zika Virus
Source: J Med Internet Res. 2019 May 13;21(5):e13090. doi: 10.2196/13090 (PMC6535980; doi:10.2196/13090)
Supplement: Multimedia Appendix 1 [file jmir_v21i5e13090_app1.docx]

## Appendix 1

- *First person:* i(’ve), me, you(’ve), we(’ve), my, our
- *Travel keywords:*
  - *General:* travel*, cancel*, airline*, *plane(s), flight*, trip*, vacation*, refund*, ticket*, honeymoon, babymoon, warn*, advisory, advisories, cruise, holiday, plan*, booking, booked, abroad, fly*, penalty, vacay, tourist, waiver, alert, waiv*, waiving, tourism, restricted, resort, hotel
  - *Airlines:* American airlines, delta, united, latamairlinesgroup, latamairlinesbrasil, aviance, azulairlines, aerolineasargentinas, jet blue, spirit airlines, avianca brasil, latam airlines argentina, latam airlines ecuador, tame, carribean airlines, boliviana de aviacion, avior airlines, surinam airways, fly jamaica, vivaclombia, viva colombia, copa, aeromexico, british airways, gol linhas aeraeas, air france, klm, air canada, iberia, lufthansa, emirates, tap air portugal, alitalia, turkish airlines, interjet, air europe, quatar airways, swiss, qantas, norwegian air uk, latin american wings, air new zealand, south african airways, air china, royal air maroc, ethiopian airlines, taca, aruba airlines, condo alaska airlines, southwest airlines, frontier airlines, allegiant air, hawaiian airlines
  - *Airline associated Twitter handles:* americanair, deltaassist, ualistheworst, latamairlines, latam chi, latam news, latamair- linesus, latam bra, latam co, azulinhasaereas, aerolineas ar, jetblue, spiritairlines, spiritairsucks, aviancabrasil, latam arg, latam ecu, tame ep, iflycarribean, caribair, caribeanairline, boabolivia, aviorairlines, avior ops, surinamairways, fly jamaica, flyjamaica air, vivacolombiaco, vivacolfansclub, copaiarlines, aeromexicousa, am escucha, british airways, voegoloficial, volargoldoficial, voegolatende, voegolri, airfrance, afnewsroom, klm uk, klmfan, klm de, klm press, klm us, airfranceklm, aircanada, acaltitude, aircanadavac, aircanadarouge, iberia en, lufthansa de,emiratessupport, tapairportugal, tap portugal, turkishairlines, tk tr, aireuropa, a4europe, aci europe, qatarairways, qrsupport, swissairlines, flyswiss, swissair, qfcustomer- care, qftravelinsider, norwegianuki, fly norwegian, vuelalaw, flyairnz, airnzaustralia, flysaa, saavacations, airchina, chinaair- linesen, ram maroc, royalairmarocuk, flyethiopian, et botswana, tacaairlines, arubaairlines, arubavln, airubaairpa, condo america, condorairline, condorcares, alaskaair, southwestair, flyfrontier, allegiant, hawaiianair
  - *Common cruise companies and related handles:* royalcarribean, royal carribean, norwegian, carnival
  - *Common travel agencies and related handles:* virgin holidays, virginholidays, thompsons holidays, thompsonsh, thompsonstravel, groupongetaways, kayak, expedia, travelocity, orbitz

Here, * indicates that this would match other words, like *traveling* or *cancelled*. Filters were case-insensitive. All major airlines in the United States were identified using [73], and all airlines with flights to South America were identified using [74].
