# Supplementary material for: Identifying Protective Health Behaviors on Twitter: Observational Study of Travel Advisories and Zika Virus
Source: J Med Internet Res. 2019 May 13;21(5):e13090. doi: 10.2196/13090 (PMC6535980; doi:10.2196/13090)
Supplement: Multimedia Appendix 2 [file jmir_v21i5e13090_app2.docx]

Appendix 2. Cross-validated F1 scores for classifiers stratified by n-gram range and percentage of features used (based on chi-square).

| Classifier | Percent used | 1-gram | 1- and 2-gram | 1-, 2-, and 3-gram | 2-gram | 2- and 3-gram | 3-gram |
| --- | --- | --- | --- | --- | --- | --- | --- |
| First person | 10 | 0.90 | 0.89 | 0.89 | 0.87 | 0.86 | 0.87 |
|  | 20 | 0.89 | 0.89 | 0.91 | 0.86 | 0.87 | 0.86 |
|  | 30 | 0.89 | 0.88 | 0.90 | 0.86 | 0.87 | 0.86 |
|  | 40 | 0.88 | 0.89 | 0.89 | 0.86 | 0.85 | 0.84 |
|  | 50 | 0.89 | 0.87 | 0.88 | 0.85 | 0.85 | 0.83 |
|  | 60 | 0.89 | 0.86 | 0.88 | 0.85 | 0.85 | 0.83 |
|  | 70 | 0.91 | 0.87 | 0.88 | 0.85 | 0.85 | 0.82 |
|  | 80 | 0.91 | 0.90 | 0.90 | 0.88 | 0.85 | 0.87 |
|  | 90 | 0.91 | 0.91 | 0.90 | 0.87 | 0.88 | 0.86 |
|  | 100 | *0.91^a^* | 0.90 | 0.90 | 0.87 | 0.88 | 0.87 |
| Travel consideration | 10 | 0.61 | 0.64 | 0.58 | 0.48 | 0.40 | 0.28 |
|  | 20 | 0.64 | 0.62 | 0.56 | 0.37 | 0.30 | 0.26 |
|  | 30 | 0.63 | 0.58 | 0.53 | 0.32 | 0.25 | 0.19 |
|  | 40 | 0.62 | 0.61 | 0.56 | 0.43 | 0.31 | 0.19 |
|  | 50 | 0.60 | 0.64 | 0.57 | 0.47 | 0.36 | 0.21 |
|  | 60 | 0.63 | 0.68 | 0.62 | 0.51 | 0.39 | 0.23 |
|  | 70 | 0.64 | 0.68 | 0.64 | 0.51 | 0.45 | 0.24 |
|  | 80 | 0.65 | 0.69 | 0.63 | 0.52 | 0.45 | 0.31 |
|  | 90 | 0.63 | 0.66 | 0.63 | 0.53 | 0.47 | 0.34 |
|  | 100 | *0.71* | 0.69 | 0.64 | 0.53 | 0.50 | 0.31 |
| Travel change | 10 | 0.71 | 0.72 | 0.71 | 0.69 | 0.66 | 0.60 |
|  | 20 | 0.72 | 0.73 | 0.74 | 0.65 | 0.69 | 0.60 |
|  | 30 | 0.69 | 0.75 | 0.71 | 0.69 | 0.68 | 0.52 |
|  | 40 | 0.75 | 0.74 | 0.73 | 0.64 | 0.66 | 0.59 |
|  | 50 | 0.76 | 0.78 | 0.75 | 0.65 | 0.59 | 0.60 |
|  | 60 | 0.70 | 0.77 | 0.75 | 0.69 | 0.62 | 0.61 |
|  | 70 | *0.79* | 0.78 | 0.73 | 0.67 | 0.68 | 0.61 |
|  | 80 | 0.72 | 0.77 | 0.73 | 0.67 | 0.74 | 0.60 |
|  | 90 | 0.68 | 0.71 | 0.69 | 0.67 | 0.74 | 0.71 |
|  | 100 | 0.67 | 0.74 | 0.74 | 0.71 | 0.71 | 0.70 |

^a^The best n-gram and percentage combination is italicized. Unigrams (1-gram) consistently outperform other n-gram combinations.
